# Prevalence of Kidney Disease in HIV-Infected and Uninfected Rwandan Women

**DOI:** 10.1371/journal.pone.0018352

**Published:** 2011-03-28

**Authors:** Christina M. Wyatt, Qiuhu Shi, James E. Novak, Donald R. Hoover, Lynda Szczech, Jules Semahore Mugabo, Agnes Binagwaho, Mardge Cohen, Eugene Mutimura, Kathryn Anastos

**Affiliations:** 1 Mount Sinai School of Medicine, New York, New York, United States of America; 2 School of Public Health, New York Medical College, Valhalla, New York, United States of America; 3 Henry Ford Health System, Detroit, Michigan, United States of America; 4 Rutgers, The State University of New Jersey, New Brunswick, New Jersey, United States of America; 5 Division of Nephrology, Department of Medicine, Duke University Medical Center, Durham, North Carolina, United States of America; 6 Institute of Human Virology, University of Maryland, Baltimore, Maryland, United States of America; 7 Ministry of Health, Kigali, Rwanda; 8 Harvard Medical School, Boston, Massachusetts, United States of America; 9 CORE Center, Cook County Bureau of Health Services, Chicago, Illinois, United States of America; 10 Women's Equity in Access to Care and Treatment, Kigali, Rwanda; 11 Montefiore Medical Center and Albert Einstein College of Medicine, Bronx, New York, United States of America; University of Nebraska - Lincoln, United States of America

## Abstract

**Background:**

In the United States, HIV-related kidney disease disproportionately affects individuals of African descent; however, there are few estimates of kidney disease prevalence in Africa. We evaluated the prevalence of kidney disease among HIV-infected and uninfected Rwandan women.

**Methods:**

The Rwandan Women's Interassociation Study and Assessment prospectively enrolled 936 women. Associations with estimated glomerular filtration rate (eGFR)<60 mL/min/1.73 m^2^ and proteinuria were assessed in separate logistic regression models.

**Results:**

Among 891 non-pregnant women with available data, 2.4% had an eGFR<60 mL/min/1.73 m^2^ (calculated by the Modification of Diet in Renal Disease equation, MDRD eGFR) and 8.7% had proteinuria ≥1+. The prevalence of decreased eGFR varied markedly depending on the estimating method used, with the highest prevalence by Cockcroft-Gault. Regardless of the method used to estimate GFR, the proportion with decreased eGFR or proteinuria did not differ significantly between HIV-infected and -uninfected women in unadjusted analysis. After adjusting for age and blood pressure, HIV infection was associated with significantly higher odds of decreased MDRD eGFR but not proteinuria.

**Conclusion:**

In a well-characterized cohort of Rwandan women, HIV infection was associated with decreased MDRD eGFR. The prevalence of decreased eGFR among HIV-infected women in our study was lower than that previously reported in African-Americans and in other Central and East African HIV populations, although there was substantial variability depending on the equation used to estimate GFR. Future studies are needed to optimize GFR estimates and to determine the impact of antiretroviral therapy on kidney disease in this population.

## Introduction

In 2008 there were an estimated 33.4 million individuals living with human immunodeficiency virus (HIV) infection. Sub-Saharan Africa bears the greatest burden of disease, with two-thirds of all HIV-infected individuals [1]. Strong genetic susceptibility to chronic kidney disease (CKD) and end-stage renal disease (ESRD) has been observed in African-Americans [2]–[4], and the risk of ESRD is as much as 30-fold higher among HIV-infected Americans of African compared to European descent [5], [6]. Epidemiological data on CKD in sub-Saharan Africa are scarce, and available data demonstrate substantial variability in CKD prevalence across different African HIV populations [7]–[10]. The regional prevalence of CKD may influence the approach to screening and monitoring of HIV-infected individuals initiating antiretroviral therapy (ART). In particular, most nucleoside/nucleotide reverse transcriptase inhibitors (NRTI) are eliminated by the kidney and may require dose adjustment in individuals with decreased glomerular filtration rate (GFR), while agents such as the NRTI tenofovir and the protease inhibitor indinavir have known nephrotoxic potential and may require more intensive monitoring in individuals with pre-existing CKD [11].

We examined enrollment data from the Rwandan Women's Interassociation Study and Assessment (RWISA), an observational cohort study of HIV-infected and uninfected Rwandan women, to determine the prevalence of kidney disease in this previously unstudied population.

## Methods

RWISA is an observational prospective cohort study investigating the effectiveness and toxicity of ART in HIV-infected Rwandan women. Nine hundred thirty-six women (710 HIV infected and 226 HIV uninfected) enrolled in 2005. Written informed consent was obtained in accordance with protocols approved by the Rwandan National Ethics Committee and the Institutional Review Board of Montefiore Medical Center, Bronx NY, USA. Inclusion criteria for RWISA included age ≥25 years, willingness to be HIV tested and return for follow-up, and presence in Rwanda in 1994. HIV-infected women were also naïve to ART at entry, *except* for exposure to single-dose nevirapine. At study entry, participants provided demographic information and medical and psychosocial history. Physical examinations and bioimpedance analysis were performed by trained research nurses. Specimens were obtained at enrollment for CD4 cell count, serum creatinine and albumin, urine pregnancy test, and dipstick urinalysis.

Blood pressure was recorded as the average of two sphygmomanometry measures. Total body fat and fat free mass were calculated using established techniques [12]. Spot urine samples were tested for the presence of protein and blood using the AimStick 10-SG Urine Reagent Strips (Germaine Laboratories, San Antonio, Texas USA) or the Humantest Combina 10 M (Wiesbaden, Belgium). Proteinuria was defined as the presence of ≥+1 protein on a single urine specimen obtained at the enrollment visit. To minimize artifact from menses or cystitis, specimens with significant hematuria (medium or large blood) were excluded (n = 21). Serum creatinine was measured by Jaffe reaction (Beckman Instrument Inc., Brea, CA, USA). Estimated glomerular filtration rate (eGFR) was calculated from a single serum creatinine value obtained at the enrollment visit, using the 4-variable Modification of Diet in Renal Disease (MDRD), CKD Epidemiology Collaboration (CKD-EPI), and Cockcroft-Gault equations [13]–[15]. Primary analyses were based on MDRD eGFR<60 mL/min/1.73 m^2^ to allow comparison with previous observational studies and with current guidelines for the diagnosis of CKD [11]. We also considered an eGFR cutoff of 50 mL/min/1.73 m^2^, the level at which dose adjustment is recommended for most NRTI. Finally, we considered the impact of eliminating the adjustment factor for black race in the MDRD and CKD-EPI equations, based on evidence that this adjustment may not perform well in African populations [16], [17]. Pregnant women (n = 16) were excluded from the current analysis due to physiologic changes in GFR and urinary protein excretion during pregnancy. To reduce the impact of outliers on the evaluation of associations between body composition and CKD prevalence, extreme weight outliers with body mass index (BMI)≥30 were also excluded (n = 29).

### Statistical analysis

Continuous and categorical variables were compared between groups using Student's T-test or Wilcoxon rank sum test and Pearson's Chi-Square or Fisher's Exact test as appropriate. Stepwise multivariable logistic regression evaluated associations with proteinuria and eGFR<60 mL/min/1.73 m^2^ among the cohort overall and in HIV-infected women in separate models. Variables tested for inclusion in the models included HIV status, age, serum albumin, BMI, lean body mass, total body fat, and systolic and diastolic blood pressure. CD4 count was assessed in HIV-infected women. As the primary predictor of interest, HIV serostatus was forced into multivariate models, while other covariates with p-value<0.05 were retained in the final models using backwards selection.

## Results

Eight hundred ninety-one non-pregnant women were included in the current analysis (677 HIV infected and 214 uninfected), including 865 with data available for estimation of GFR and 739 with eligible urine specimens for assessment of proteinuria (Table 1). HIV-infected women were younger than HIV-uninfected women (median age 34 *versus* 43 years, p<0.01). Self-reported diabetes and hypertension were rare, with a trend towards more hypertension among HIV-uninfected women. Median weight was 52 kg and median BMI was 20.9 kg/m^2^, with no significant difference between HIV-infected and HIV-uninfected women.

**Table 1:** Clinical and demographic variables stratified by HIV status.

	HIV-infected(n = 677)Median (IQR)Or n (%)	HIV-uninfected(n = 214)Median (IQR)Or n (%)	p-value^b^
Age (years)	34 (30–39)	43 (34–50)	<0.01
Self-reported diabetes mellitus	3 (0.5%)(n = 671)^a^	1 (0.5%)(n = 204)^a^	1.00
Self-reported hypertension	32 (4.8%)(n = 671)^a^	17 (8.3%)(n = 204)^a^	0.06
Weight (kg)	52 (46–58)	52 (46–61)	0.23
Body mass index (kg/m^2^)	20.9 (19.0–23.3)	20.5 (18.5–23.3)	0.23
Lean body mass (kg)	40.3 (37.3–42.9)(n = 570)^a^	41.4 (38.6–44.7)(n = 204)^a^	<0.01
Total body fat (kg)	11.8 (7.8–16.5)(n = 568)^a^	10.5 (6.6–18.1)(n = 204)^a^	0.47
Systolic blood pressure (mmHg)	119 (110–122.5)	120 (110–125)(n = 213)^a^	0.019
Diastolic blood pressure (mmHg)	70 (65–78)	72 (68.5–80)(n = 212)^a^	<0.01
CD4 cell count (cells/µl)	256 (163–360)(n = 675)^a^	----	----
WHO Stage 4 HIV (with weight loss)	281 (41.5%)	----	----
Serum albumin (g/dL)	3.5 (3.1–3.8)(n = 659)^a^	4.0 (3.6–4.2)(n = 209)^a^	<0.01
Serum creatinine (mg/dL)	0.92 (0.82–1.02)(n = 659)^a^	0.85 (0.75–0.96)(n = 206)^a^	<0.01
eGFR (mL/min/1.73 m^2^)	90.0 (78.4–103.9)	94.5 (82.4–109.3)	<0.01
eGFR<60 mL/min/1.73 m^2^	18 (2.7%)	3 (1.5%)	0.44
Proteinuria (urine protein ≥1+)	55 (9.0%)(n = 614)^a^	9 (7.2%)(n = 125)^a^	0.60

a For variables with missing data, the number of women with available data is reported.

b Fisher's Exact test for categorical and Wilcoxon two-sample test for continuous variables.

IQR: interquartile range; WHO, World Health Organization Clinical Stage of HIV; eGFR: estimated glomerular filtration rate by 4-variable Modification of Diet in Renal Disease (MDRD) equation.

Overall, 21 women (2.4%) had an MDRD eGFR<60 mL/min/1.73 m^2^ and 64 women (8.7%) had proteinuria ≥1+ (Table 1). The prevalence of decreased eGFR varied substantially depending on the estimating method used, with a nearly 10-fold higher prevalence of eGFR<60 mL/min/1.73 m^2^ when the Cockcroft-Gault equation was employed (Table 2). Similar patterns were observed for eGFR<50 mL/min/1.73 m^2^ and when the adjustment factor for black race was eliminated from the MDRD and CKD-EPI equations (Table 2). There was only one participant with both proteinuria and decreased MDRD eGFR, a 50-year-old HIV-infected woman with a CD4 cell count of 249 cells/m^3^. Although there were 20 women with both proteinuria and Cockcroft-Gault eGFR<60 mL/min, there was no significant association between proteinuria and decreased eGFR regardless of the method used to estimate GFR (data not shown).

**Table 2:** Prevalence of decreased glomerular filtration rate stratified by HIV status and estimating equation.

	HIV-infected(n = 659)n (%)	HIV-uninfected(n = 206)n (%)	p-value
eGFR<60 mL/min/1.73 m^2^			
MDRD	18 (2.7%)	3 (1.5%)	0.44
MDRD unadjusted for race	97 (14.7%)	22 (10.7%)	0.16
CKD-EPI	16 (2.4%)	2 (1.0%)	0.27
CKD-EPI unadjusted for race	52 (7.9)	14 (6.8%)	0.66
Cockcroft-Gault (mL/min)	166 (25.2%)	50 (24.3%)	0.79
eGFR<50 mL/min/1.73 m^2^			
MDRD	4 (0.6%)	0 (0.0%)	0.58
MDRD unadjusted for race	20 (3.0%)	3 (1.5%)	0.32
CKD-EPI	4 (0.6%)	0 (0.0%)	0.58
CKD-EPI unadjusted for race	10 (1.5%)	1 (0.5%)	0.47
Cockcroft-Gault (mL/min)	60 (9.1%)	20 (9.7%)	0.79

eGFR: estimated glomerular filtration rate; MDRD: 4-variable Modification of Diet in Renal Disease equation; CKD-EPI: Chronic Kidney Disease Epidemiology Consortium equation.

The proportion of women with decreased eGFR or proteinuria did not differ significantly between HIV-infected and -uninfected women, regardless of the equation used to calculate eGFR. However, after adjusting for age, blood pressure, body composition, and serum albumin (Table 3), HIV infection was independently associated with decreased MDRD eGFR (adjusted OR 8.9, 95% CI 1.6–50.5), but not with the presence of proteinuria (adjusted OR 1.7; 95% CI 0.7–4.1). Higher diastolic blood pressure was also independently associated both with decreased MDRD eGFR and with proteinuria, while older age was associated with decreased MDRD eGFR in the overall cohort. Among HIV-infected women, older age remained a predictor of decreased MDRD eGFR, while higher diastolic blood pressure and lower serum albumin were independently associated with the presence of proteinuria (Table 4). Although the proportion with either proteinuria or decreased MDRD eGFR was highest among HIV-infected women with CD4 cell count <200 (15% versus 9% in HIV-infected women with CD4 cell count ≥200, p = 0.05), CD4 cell count was not significantly associated with decreased MDRD eGFR or with proteinuria in adjusted analyses. There was no association of decreased MDRD eGFR or proteinuria with measures of body composition, including BMI, lean body mass, or total body fat.

**Table 3:** Univariate and multivariate associations with kidney disease in all participants.

	eGFR<60^a^Unadjusted OR	Adjusted OR	Proteinuria^b^Unadjusted OR	Adjusted OR^c^
HIV Status (1 for +, 0 for −)	1.90 (0.55–6.52)	8.91 (1.57–50.49)*	1.27 (0.61–2.64)	1.66 (0.67–4.12)
Age (years)	1.63 (1.07–2.50)*	2.20 (1.31–3.68)**	1.33 (1.00–1.77)*	1.33 (0.96–1.86)
Body mass index (per kg/m^2^)	1.10 (0.74–1.63)		0.87 (0.68–1.11)	
Lean body mass (per kg)	1.34 (0.87–2.06)		0.90 (0.67–1.21)	
Total body fat (per kg)	1.17 (0.86–1.60)		0.88 (0.71–1.08)	
Systolic blood pressure	1.24 (0.94–1.65)		0.99 (0.79–1.25)	
Diastolic blood pressure	1.49 (1.01–2.21)*	1.68 (1.08–2.63)*	1.36 (1.02–1.82)*	1.42 (1.05–1.91)*
Serum albumin (per g/dL)	0.91 (0.48–1.70)		0.69 (0.48–0.99)*	0.64 (0.43–0.94)*
Serum creatinine (per mg/dL)			1.51 (0.39–5.86)	

Note: * for p<0.05, ** for p<0.01 and *** for p<0.001.

a Estimated glomerular filtration rate calculated using the 4-variable Modification of Diet in Renal Disease Equation.

b Urine protein 1+ or greater.

c Backwards stepwise selection with a p = 0.05 to remain and HIV serostatus forced in.

**Table 4:** Univariate and multivariate associations with kidney disease in HIV-infected participants.

	eGFR<60^a^Unadjusted OR	Adjusted OR	Proteinuria^b^Unadjusted OR	Adjusted OR^c^
Age (years)	2.20 (1.27–3.81)**	2.20 (1.27–3.81)**	1.38 (0.95–2.00)	
Body mass index (per kg/m^2^)	0.85 (0.54–1.33)		0.98 (0.75–1.27)	
Lean body mass (per kg)	1.22 (0.75–1.98)		0.96 (0.70–1.33)	
Total body fat (per kg)	1.02 (0.71–1.47)		0.98 (0.78–1.22)	
Systolic blood pressure	0.89 (0.57–1.39)		1.02 (0.77–1.33)	
Diastolic blood pressure	1.08 (0.58–2.03)		1.88 (1.28–2.76)**	1.95 (1.33–2.88)***
CD4 cell count (per 100 cells/µl)	0.91 (0.67–1.23)		0.82 (0.67–0.99)*	
Serum albumin (per g/dL)	1.00 (0.50–1.99)		0.60 (0.40–0.88)**	0.56 (0.37–0.83)**
Serum creatinine (per mg/dL)			1.22 (0.27–5.44)	

Note: * for p<0.05, ** for p<0.01 and *** for p<0.001.

a Estimated glomerular filtration rate calculated using the 4-variable Modification of Diet in Renal Disease Equation.

b Urine protein 1+ or greater.

c Backwards stepwise selection with a p = 0.05 to remain.

## Discussion

In this well-characterized cohort of Rwandan women, HIV infection was independently associated with decreased MDRD eGFR in adjusted, but not unadjusted, analysis. The prevalence of decreased MDRD eGFR among HIV-infected women in this study was lower than that observed in previous studies in HIV-infected African-Americans [5], [18] and in other Central and East African populations [7], [9], [10], [19], while the prevalence of proteinuria was similar to that previously reported among HIV-infected individuals in Kenya and South Africa [7], [20]. Consistent with other recent reports from sub-Saharan Africa, the prevalence of decreased eGFR varied substantially depending on the estimating method used [16], [17].

Both HIV-associated nephropathy (HIVAN) and HIV-related ESRD are known to disproportionately affect African-Americans [5], [6]. With the recent discovery of a locus on chromosome 22 that is associated with genetic susceptibility to HIVAN and other forms of CKD and ESRD among African-Americans [2]–[4], there is increasing concern about the burden of HIV-related CKD in sub-Saharan Africa [21]. Available data suggest substantial regional variability in the prevalence of HIV-related CKD. The highest burden has been observed in West Africa, consistent with the predominant ancestry of the genetically susceptible African-American population [8].

With expanding access to ART across Africa, including the use of agents with nephrotoxic potential, the local prevalence of HIV-related CKD may be a valuable determinant of the need to screen and monitor for CKD in individuals initiating ART. The Development of ART in Africa Trial (DART) randomized adults initiating ART in Uganda and Zimbabwe to routine laboratory monitoring or clinically driven monitoring, with laboratory data collected for all subjects but only made available to providers in the clinically driven monitoring arm when requested based on clinical signs and symptoms [22]. There was no significant difference in renal adverse events between the two monitoring strategies, supporting current World Health Organization (WHO) guidelines, which do not advocate universal laboratory screening for CKD prior to ART initiation in resource-limited settings [23]. Nonetheless, it is not known whether a clinically driven screening and monitoring strategy can be safely adopted throughout Africa, highlighting the need to investigate the prevalence of CKD and other relevant comorbidities in previously understudied populations.

The relatively low prevalence of decreased MDRD eGFR observed in Rwandan women would support the safety of WHO recommendations to forgo universal pre-ART CKD screening in this population [23]. In contrast, the Cockcroft-Gault equation identified evidence of decreased kidney function in more than one-quarter of HIV-infected women, and nearly 10% would have required dose reduction of NRTI's including tenofovir. This striking difference in the performance of different GFR estimates has been reported in other African populations [7], [10], [24], [25], and has been attributed to differences in body composition compared to the North American populations in which the equations were derived (Table 5). Our primary analyses were based on MDRD eGFR to allow comparison with previous observational studies and current guidelines for the diagnosis of CKD [11]; however, future studies are needed to validate or optimize GFR estimates for use in African populations. With increasing use of tenofovir as first-line therapy, these studies may influence guidelines for CKD screening and monitoring in patients initiating ART in resource-poor settings.

**Table 5:** Comparison of RWISA participants to the MDRD, CKD-EPI, and Cockcroft-Gault study populations.

	Mean Weight	Mean BSA	Mean BMI	Mean Age	Female	Black race
**MDRD**	80 kg	1.91 m^2^	27 kg/m^2^	51 years	40%	<12%
**CKD-EPI**	82 kg	1.93 m^2^	28 kg/m^2^	47 years	43%	32%
**Cockcroft-Gault**	72 kg	NA	NA	Range 18–92 years	NA	NA
**RWISA**	53 kg	1.52 m^2^	21 kg/m^2^	37 Years	100%	100%

MDRD: Modification of Diet in Renal Disease Equation; CKD-EPI: Chronic Kidney Disease Epidemiology Consortium Equation; BSA: body surface area; BMI: body mass index.

Prior studies in Central and East Africa have reported a higher prevalence of decreased MDRD eGFR among ART-naïve individuals than we observed in the current study [7], [10], [19]. Several of those studies included patients with more advanced HIV disease, as evidenced by lower CD4 cell count or symptomatic disease [9], [10], [24]. In contrast, the prevalence of proteinuria observed in our population was similar to that reported in studies from Kenya and South Africa [7], [20]. We have previously reported increased mortality associated with proteinuria among HIV-infected women in the United States [26]–[28], and future studies should investigate whether proteinuria is also a predictor of adverse outcomes among HIV-infected individuals in Africa.

In addition to the lack of validated GFR estimates for use in an African population, this study has several other important limitations. RWISA enrolled a cohort of HIV-infected women with a broad range of CD4 count and clinical disease, and prevalence estimates derived from this study may not be generalizable to Rwandan men and older women or to individuals with very advanced HIV disease. In addition, we were limited by the use of a single serum creatinine and a single urinalysis to identify women with kidney disease. Guidelines for CKD diagnosis require documentation of decreased eGFR or proteinuria for at least 3 months [11], and application of these guidelines would likely result in a lower estimated prevalence of CKD than we have reported. Nonetheless, repeated measures are rarely feasible prior to ART initiation in resource-poor settings, and future guidelines will likely rely on at most a single laboratory result to drive decision-making in this setting.

In a well-characterized cohort of Rwandan women, HIV infection was associated with decreased MDRD eGFR but not with the presence of proteinuria. The prevalence of decreased eGFR among HIV-infected women was lower than that reported in previous studies in Central and East Africa, supporting the WHO's recommended strategy of targeted pre-ART screening for CKD in this setting [23]. Proteinuria was observed more commonly in our population. Although there was no correlation between the presence of proteinuria and decreased eGFR at the time of enrollment, future studies should investigate whether proteinuria is a useful non-invasive marker of risk for nephrotoxicity in African women initiating ART. Importantly, this study also highlights the need for future research to validate and optimize GFR estimates for use in African populations.
